# Survivin Small Molecules Inhibitors: Recent Advances and Challenges

**DOI:** 10.3390/molecules28031376

**Published:** 2023-02-01

**Authors:** Najah Albadari, Wei Li

**Affiliations:** Department of Pharmaceutical Sciences, College of Pharmacy, University of Tennessee Health Science Center, Memphis, TN 38163, USA

**Keywords:** survivin, apoptosis, mitosis, chemoresistance, survivin small molecules inhibitors

## Abstract

Survivin, as a member of the inhibitor of apoptosis proteins (IAPs) family, acts as a suppressor of apoptosis and plays a central role in cell division. Survivin has been considered as an important cancer drug target because it is highly expressed in many types of human cancers, while it is effectively absent from terminally differentiated normal tissues. Moreover, survivin is involved in tumor cell resistance to chemotherapy and radiation. Preclinically, downregulation of survivin expression or function reduced tumor growth induced apoptosis and sensitized tumor cells to radiation and chemotherapy in different human tumor models. This review highlights the role of survivin in promoting cellular proliferation and inhibiting apoptosis and summarizes the recent advances in and challenges of developing small-molecule survivin inhibitors.

## 1. Introduction

Programmed cell death serves fundamental functions during mammalian tissue development. Apoptosis is a highly regulated and controlled process and represents one form of programmed cell death. Defective and insufficient apoptosis processes can result in uncontrolled cell proliferation and cancer. Apoptosis is initiated by activating either the intrinsic or the extrinsic pathway, and it is executed by caspases. The intrinsic pathway is activated by endogenous stress signals or irradiation, which depends on the release of Cytochrome c (Cyt-c) from the mitochondria. In contrast, the extrinsic pathway (also known as the death receptor pathway) is mitochondrion-independent and is activated by extracellular ligands binding to cell-surface death receptors [1]. Apoptosis can be blocked by endogenous proteins such as the inhibitors of apoptosis proteins (IAPs). IAP proteins were first identified in baculoviruses, where they could inhibit the host’s defensive apoptotic response to infected insect cells and enhance viral replication [2]. Subsequently, several cellular IAP homologs were found in diverse organisms, including vertebrates, insects, and yeasts [3,4]. The human IAPs are a family of eight structurally and functionally related proteins. All IAP family members have one to three copies of a baculovirus IAP repeat (BIR), a domain with ~70 amino acids, which is the main mediator of the antiapoptotic function. Survivin is the smallest member of the IAPs and has only one BIR domain [5]. Survivin is a multi-tasking protein that is both essential for mitosis and can inhibit apoptosis. It has gained significant attention as a potential therapeutic target for cancer partially because it is expressed only in most rapidly dividing cells, such as cancer cells, while its expression is very low in differentiated normal cells. In addition, survivin expression correlates positively with chemoresistance, radiation insensitivity, and poor patient prognosis. Moreover, survivin plays a role in promoting tumor cell survival and cancer metastasis. This review highlights our understanding of the structure, expression, and functions of survivin and summarizes the recent advances in developing small-molecule survivin inhibitors for potential cancer therapy.

## 2. Structure and Cellular Functions of Survivin

**Figure 1:**
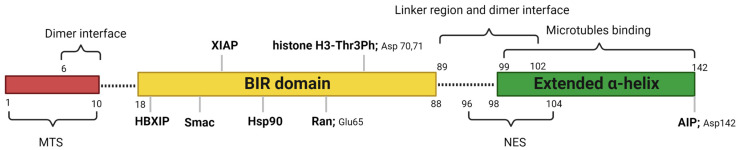
Critical features of survivin structure and its interaction sites with its key partners. Survivin has a single BIR domain (18–88 aa), a linker region (89–102 aa), and an extended α helix (99–142 aa) in its C-terminal. The first 10 amino acids in its N-terminus are proline-rich and represent the MTS for mitochondrial importation of survivin. The BAR domain is functionally significant for survivin interactions with Smac, Ran, XIAP, HBXIP, and histone 3 of the centromeric chromatin. The linker region mediates the dimerization along with residues 6–10 in the N-terminal region of the survivin. Borealin replaces one survivin monomer through the dimer interface to form a survivin–borealin heterodimer. The C-terminal of survivin, the N-terminal of borealin, and INCENP form the triple helix unit as part of CPC. The survivin helical region has tubulin- and AIP-binding sites. Survivin has an NES between the BIR domain and the C-terminal helix, which is masked by the homodimerization interface. Moreover, survivin has a non-classical bipartite NES in the C-terminal. This figure was created with BioRender.com.

Human survivin is a small protein with a molecular weight of 16.5 kDa and contains 142 amino acid residues. Structurally, human survivin closely resembles the BIR-containing proteins from yeasts and C. elegans [3,6]. Human survivin has a signal BIR domain (aa 18–88) in the N-terminal, followed by a linker region (aa 89–102) and an extended α-helix (aa 98–142) in the C-terminal (Figure 1) [6]. The BIR domain is stabilized by a zinc finger created by four amino acids: Cys57, Cys60, His77, and Cys84 [6]. Survivin exists in the body as both a monomer and a homodimer. Survivin monomers form a homodimer through interactions located mainly in the linker region and residues 6–10 in the N-terminal region of the BIR domain [5,7]. Survivin homodimerization for function is not always needed, since both the survivin monomer and dimer are functional. While most IAP proteins are predominantly cytosolic, survivin has been found in the cytoplasm, nucleus, mitochondria [8], exosomes [9], outer surface of the cell membrane, and extracellular matrix. Survivin possesses a dual function in the body: protection from apoptosis and regulation of cell division [10]. Recent reports also suggested that survivin is involved in autophagy [11], angiogenesis [12], and stemness [13].

### 2.1. Cytoplasmic and Mitochondrial Survivin 

**Figure 2:**
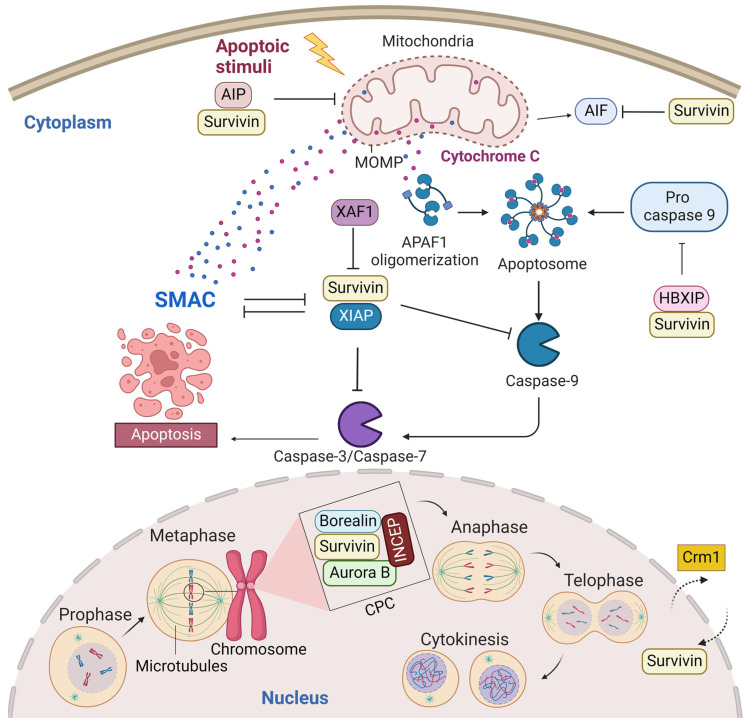
Survivin is an antiapoptotic protein and essential mitotic protein. Cytoplasmic survivin and mitochondrial survivin inhibit apoptosis, while nuclear survivin regulates cell division. Crm1 mediates the exportation of survivin from the nucleus to the cytoplasm. Survivin plays a significant role in inhibiting the intrinsic apoptotic pathway. The intrinsic apoptotic pathway is activated by endogenous stress signals or irradiation and depends on the release of Cyt-c from the mitochondria. Cyt-c binds with Apaf-1 and ATP, which bind to pro-caspase-9 and create a protein complex known as an apoptosome. Pro-caspase-9 is cleaved to its active form of caspase-9 by the apoptosome, which in turn activates the effector caspase-3/6/7, resulting in cell apoptosis. Survivin stabilizes XIAP and blocks Smac from antagonizing XIAP. Survivin–XIAP complex blocks caspase-9 and inhibits apoptosis. Survivin-HBXIP also suppresses pro-caspase-9 and blocks the activation of caspase-9. The survivin–AIP complex stabilizes survivin and promotes its anti-apoptotic function in the mitochondria. Mitotic survivin is part of the CPC and directs Aurora-B kinase to centromere during mitosis. This figure was created with BioRender.com.

A predominant cytoplasmic and mitochondrial localization of survivin is essential for its anti-apoptotic activity [14]. Many reports showed its upregulation in the cytoplasmic expression in cancer cells [15]. Survivin is exported from the nucleus to the cytoplasm by chromosome region maintenance 1 (Crm1, also known as exportin-1) [16]. Survivin–Crm1 interaction is mediated by a centrally located leucine-rich nuclear export sequence (NES) that is placed between the BIR domain and the C-terminal helix of survivin and a non-classical bipartite NES in the C-terminus [17,18]. The central NES is primarily active in survivin monomers because it is partially masked by the homodimerization interface [19]. Survivin physically associates with the X-linked inhibitor of apoptosis protein (XIAP), another member of the IAPs, through its BIR domain, protects it from ubiquitination, and enhances its stability. A stabilized survivin–XIAP complex suppresses caspase-9 activity and blocks apoptosis in vivo (Figure 2) [20]. However, XIAP-associated factor 1 (XAF1), a nuclear protein that binds to XIAP and suppresses its anti-caspase activity, reverses the inhibition of the ubiquitination of XIAP by survivin and activates the XIAP E3 ligase to target and promote survivin degradation [21,22]. Survivin also augments the anti-apoptotic function of XIAP via another mechanism, where survivin binds to the second mitochondria-derived activator of caspases (Smac). Smac, also known as DIABLO, is a mitochondrial protein that is released into the cytosol following the increase in the mitochondrial outer membrane permeability (MOMP) during apoptosis. Smac antagonizes IAPs, including XIAP, cellular inhibitor of apoptosis protein 1 (CIAP-1), and cellular inhibitor of apoptosis protein 2 (CIAP-2), and promotes Cyt-c-dependent caspase activation [23]. Survivin sequesters Smac away from other IAPs and protects the cell from mitochondria-regulated apoptosis [24]. Survivin interacts with Smac via its BIR domain, thereby freeing XIAP and allowing it to block caspases without being antagonized [24]. The BIR domain of survivin is also necessary for binding with the hepatitis B virus X-interacting protein (HBXIP, also known as LAMTOR5). HBXIP operates as a cofactor for survivin and allows the HBXIP–survivin complex to bind pro-caspase-9 and preclude pro-caspase-9 recruitment to activated apoptotic protease activating factor-1 (Apaf-1) and, thus, suppress activation of caspase-9 [25]. In addition to caspase inhibition, survivin has been reported to inhibit caspase-independent apoptosis. Survivin blocks the release of apoptosis-inducing factor (AIF), the primary mediator of caspase-independent apoptosis, from the mitochondrial intermembrane space (IMS) and its nuclear translocation [26]. In response to different apoptosis stimuli, AIF translocates from IMS to the nucleus, causing DNA fragmentation and chromatin condensation [26,27]. Survivin importation into mitochondria is directed through the mitochondrial targeting sequence (MTS) (1–10 aa) located in its N-terminus [28]. In addition, survivin directly associates with aryl hydrocarbon receptor-interacting protein (AIP) [29]. This interaction is mediated by the survivin carboxyl terminus coiled coil and three tetratricopeptide motifs located in the carboxyl-terminal end of AIP. Aspartic acid 142 (Asp142), the last amino acid in survivin, plays a critical role in AIP recognition [29]. The survivin–AIP complex stabilizes survivin levels and enhances its anti-apoptosis function in the mitochondria [29]. Survivin also interacts with heat shock protein 90 (Hsp90), where Hsp90 preserves survivin stability in vivo [30]. Hsp90 association with survivin involves the ATPase domain of Hsp90 and the survivin BIR domain. Disruption of the survivin–Hsp90 interaction results in the proteasomal degradation of survivin and mitochondrial-dependent apoptosis [30]. 

### 2.2. Nuclear Survivin

Nuclear survivin plays a pivotal role in the coordination of mitosis and cytokinesis. Survivin localization in the nucleus, thus far, is thought to occur by passive diffusion, as no classical nuclear localization signal (NLS) exists within the protein. Some reports showed that the nuclear localization of survivin is proapoptotic and increases the susceptibility of cancer cells to conventional chemotherapy and radiation treatment. For example, Colnaghi et al. showed that mutant survivin accumulating in the nucleus could no longer protect cells against the ionizing radiation or apoptosis induced by the TNF-related apoptosis-inducing ligand [14,31]. Moreover, when Connell et al. artificially forced wild-type human survivin expression in the nucleus, they observed that the nuclear localization of survivin prevented it from acting as an inhibitor of apoptosis [32]. However, other reports showed that nuclear survivin is antiapoptotic and associated with poor prognosis in various tumor types [15,33,34]. This contradiction in data regarding the role of nuclear survivin as a predictor for prognosis may be tumor-type-specific and/or due to the variable criteria used to classify a tumor as nuclear survivin or cytoplasmic survivin. Nuclear survivin has a highly dynamic and characteristic localization pattern during mitosis. The localization pattern of survivin during mitosis is typical for chromosomal passenger proteins. In fact, the survivin monomer can interact with the chromosomal passage protein, Borealin, during mitosis [35]. Borealin uses the same survivin–survivin dimerization interface to interact and replace one survivin monomer to form a survivin–borealin heterodimer [35]. Survivin, together with borealin and the inner centromere protein (INCENP) form the non-enzymatic regulatory components of the chromosomal passenger complex (CPC), an essential mitotic complex (Figure 2). The N-termini of borealin and survivin associate with INCENP to form a tight three-helical bundle and create a single structural unit [35]. The association of the core “passenger” proteins controls the activity and localization of the CPC enzymatic component, the Aurora B kinase, both temporally and spatially [36]. Survivin targets the CPC to the centromeres during prometaphase, ensuring that chromosomes are correctly aligned before they are segregated at anaphase. The BIR domain of survivin (residues Asp70 and Asp71) recognizes histone 3 in centromeric chromatin that has been phosphorylated at Thr3 by the haspin kinase [37,38]. Survivin binds to the N-terminal tail of the histone H3 carrying the Thr3 phosphorylation mark (Thr3ph) [37,38]. During prometaphase, survivin is phosphorylated at Thr117 by the Aurora B kinase to ensure that its association with the centromeres remains dynamic until all chromosomes have oriented [39,40,41,42]. During the metaphase–anaphase transition, survivin dissociates from the centromere and tethers at the spindle midzone, while the sister chromatids migrate to the poles. During telophase, survivin is located in the midbodies at the intercellular bridge and is degraded after cytokinesis. During cytokinesis, survivin delineates the cleavage plane prior to actomyosin recruitment [43]. 

Most studies indicate a close association between survivin and microtubules. Survivin was shown to bind to the polymerized microtubules of the mitotic spindle and the midzone [44], to centrosomes [39,43], or to kinetochores [45]. The binding site was hypothesized to be in the survivin helical region [7]. Survivin also regulates the microtubule dynamics and nucleation, as Rosa et al. demonstrated: depletion of survivin increased both the amount of microtubules nucleation and the incidence of microtubule catastrophe. In contrast, its overexpression reduced the microtubules’ nucleated by centrosomes and suppressed the microtubule dynamics in mitotic spindles and bidirectional growth of microtubules in midbodies during cytokinesis [46]. It is worth mentioning that these survivin effects on microtubules are independent of Aurora B expression or activity [46]. Survivin suppresses microtubule dynamics through its interaction with Ran, which promotes the delivery of the Ran effector molecule, the targeting protein for Xenopus kinesin-like protein 2 (TPX2), to microtubules for proper spindle formation [47]. The mitotic spindle is a bipolar-microtubule-based structure that segregates chromosomes during the cell cycle. Spindle assembly and chromosome segregation depend on the function of various microtubule motor proteins, such as microtubule-associated proteins (MAPs), which are required to regulate microtubule dynamics and the other molecules, such as the small GTPase Ran, involved in microtubule polymerization during mitosis [47]. TPX2 is a microtubule-associated protein that acts as a spindle assembly factor (SAF), mediates the binding of the COOH-terminal domain of Xenopus kinesin-like protein 2 to microtubules, and colocalizes with spindle microtubules in the M-phase in vivo and in vitro [48,49]. Survivin physically interacts with Ran via a discrete binding interface centered on Glutamic acid 65 (Glu65) in survivin [47]. Ran also regulates the Crm1–survivin/cargoes interaction, where cargoes’ binding and the release of Crm1 are controlled by the asymmetric distribution of the two nucleotide states of Ran, the so-called RanGTP gradient. The Ran guanine nucleotide exchange factor (RanGEF), RCC1, facilitates Ran binding to Crm1. Upon export to the cytoplasm, the trimeric exportin/RanGTP/substrate complex is disassembled by the RanGTP hydrolysis induced by the RanGTPase-activating protein (RanGAP) and RanGTP-binding protein 1 (RanBP1) [50].

## 3. Expression and Isoforms of Survivin

**Figure 3:**
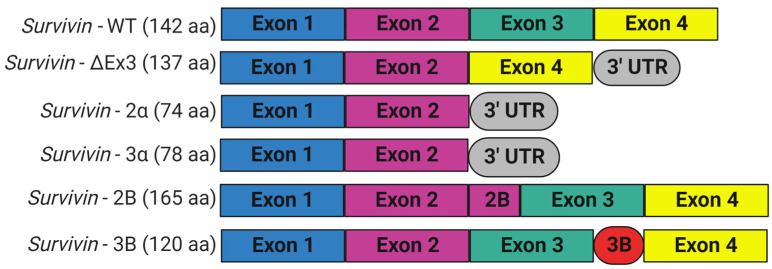
Schematic representation of alternative splice variants of survivin encoded by BIRC5 gene. Six different variants were identified. The wild-type survivin with four exons: survivin-ΔEx3 lacks exon 3 and shows a frameshift with the extension of the reading frame into the open reading frame of the 3′ untranslated region; survivin-2α and survivin-3α have exon 1 and 2; survivin-2B has an additional exon (exon 2B) inserted between exon 2 and 3; and survivin-3B has a novel exon (exon 3B) flanked by exon 3 and 4. This figure was created with BioRender.com.

Survivin is scarcely expressed in resting adult tissue yet is found in adult myeloid stem cells, adult marrow, umbilical cord blood CD34+ cells, peripheral blood mononuclear cells, and T lymphocytes; during embryogenesis; and in most human cancers [6,51,52]. Survivin is required for mitosis during development, since embryos with homozygous general deletion of survivin are embryonically lethal 4.5 days before post-coitum [43,53]. Survivin null mouse embryos showed degenerated blastomeres, micronuclei formation, variable nuclear sizes, irregular nuclear morphology, multinucleation, the absence of normal mitotic spindle structures and intercellular midbodies, reduced microtubule networks around the cells, and bundling of microtubules [43]. Consistent with its role in controlling mitotic progression, survivin levels are regulated in a cell-cycle-dependent fashion. Normally, survivin is maximally expressed during the G2/M phase of the cell cycle, where it associates with the mitotic spindle microtubules and performs functions essential for chromosome segregation and cytokinesis [44]. Survivin presents at very low levels in G1 and S phases, since it is ubiquitylated and degraded after mitosis by the 26S proteasome [54]. Survivin is preferentially degraded in the nucleus in a cdh1/APC-dependent manner [32]. The anaphase-promoting complex or cyclosome (APC/C), an E3 ubiquitin ligase, marks survivin for degradation by the 26S proteasome, to trigger the transition from metaphase to anaphase. However, when overexpressed, as it is in cancer cells, survivin is present in interphase and shuttles between the cytoplasm and nucleus. The BIRC5 gene encodes human survivin, so it is also known as a baculoviral inhibitor of apoptosis repeat-containing 5 (BIRC5). BIRC5 represents one of 40 genes to be expressed at elevated levels in all cancer tissues but not in normal cells [55]. Human BIRC5 is mapped to chromosome 17q25, and its promoter possesses a canonical CpG island and numerous Sp1 sites but no TATA box with cell cycle-regulatory sequences (cell-cycle-dependent element (CDE)/cell-cycle gene homology region (CHR)), which ensures its cell-cycle-dependent expression [56]. Moreover, the survivin promoter has multiple sites for binding prooncogenic transcription factors, including those that may be responsible for its differential expression in normal and cancer tissues [57,58]. BIRC5 has four exons and three introns, and its alternative transcription gives rise to six different transcripts, survivin, survivin-ΔEx3, survivin-2B, survivin-3B, survivin 2α, and survivin-3α, with additional variants found in the Est database (Figure 3) [6,59]. Survivin is the predominant (mature) wild-type form, and it is derived from exons 1–4 [60,61,62]. Survivin-ΔEx3 (137 amino acids) lacks exon 3; thus, its BIR domain is truncated at amino acid position 74, and it has a characteristic frameshift in the translation in its carboxyl terminus [61]. However, Survivin 2α (74 amino acids) contains the coding sequences from exon 1,2 and one additional amino acid before termination, and it lacks the entire carboxy-terminal coiled-coil domain [63]. Survivin-3α is also a truncated variant, similar to survivin-2α, and is not widely studied [64]. Survivin-2B (165 amino acids) retains a part of intron 2 as a cryptic exon that creates an additional and alternative exon named 2B that encodes the insertion of 23 additional amino acids into the BIR domain at essentially the same position (amino acid 74), where the BIR domain of survivin-ΔEx3 is truncated [61]. Survivin-3B (120 amino acids) is coded by exons 1,2, and 3. It consists of the N-terminal 113 amino acids of survivin and seven new amino acid sequences at the C-terminal tail encoded by exon 3B from intron 3 of survivin [62]. It also contains the BIR domain but lacks the carboxyl-terminal coiled-coil domain [62]. However, not all variants have been unambiguously shown to be expressed in vivo [65]. In addition, there are conflicting reports regarding the biological functions of survivin splice variants, as they may undergo homo-/heterodimerization, particularly with wild-type survivin. Recent studies looked at their potential contributions to disease and their significance as biomarkers and diagnostic tools in cancer [60]. While the expression levels of survivin and survivin-ΔEx3 correlate with tumor aggressiveness and resistance to therapy, survivin-2B and survivin-2α were reported to be cytoprotective and proapoptotic [61,63,66]. However, Knauer and colleagues reported that only survivin-3B, among the survivin isoforms, protected cells against cisplatin- or irradiation-induced apoptosis [65].

## 4. Targeting Survivin for Cancer Therapy 

**Figure 4:**
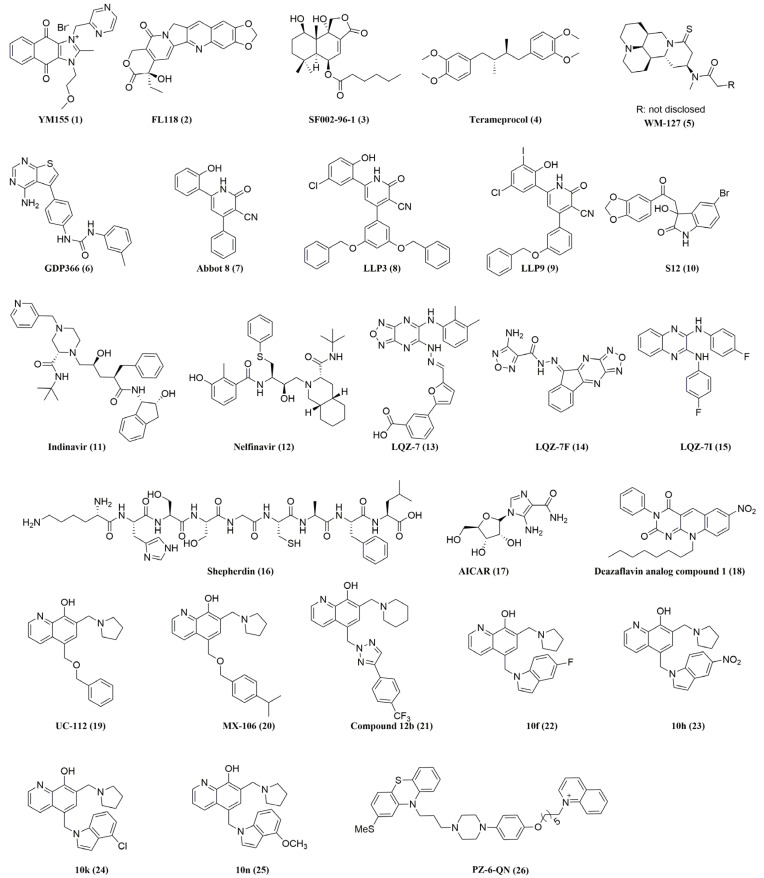
The chemical structures of survivin inhibitors reviewed in this article are shown. This figure was created using ChemDraw (version 21).

Survivin involvement in nearly every aspect of cancer dictates the pursuit of anti-survivin cancer therapies [67]. Survivin has also been considered a potential cancer drug target because of its dramatic dysregulation of expression between normal adult tissues and malignant tissues. Overexpression of survivin has been implicated in ovarian cancer [68,69], colorectal cancer [70], breast cancer [71], prostate cancer [72], gastrointestinal cancer [73], lymphoma [74,75], acute myeloid leukemia (AML) [76,77], and chronic myeloid leukemia (CML) [78,79]. The aberrant high expression of survivin in cancers is a predictive of poor clinical outcome, contributes to both radiotherapy [80] and chemotherapy resistance [81,82,83], and correlates with relapse in various cancers such as bladder cancer [84], non-small cell lung cancer [85], gall bladder carcinoma [86], locally advanced rectal cancer [87], renal cell carcinoma [88], and breast cancer [89,90]. Suppressing survivin activity can sensitize tumors to conventional therapies and help overcome multidrug resistance [91,92,93,94]. Downregulation of survivin expression by antisense oligonucleotides [95] and small interfering (si)RNAs inhibited cancer cell proliferation, promoted cell apoptosis, and enhanced chemosensitivity [96,97]. Knockout of the survivin gene by CRISPR/Cas9 can also impede cancer development [98,99]. Survivin knockdown and knockout studies, along with its expression profile in cancer and its clinical relevance, have clearly validated survivin as a target in cancer therapy. In this context, different approaches to counteract survivin in tumor cells have been reported in the literature. These antisurvivin approaches can be classified into six categories: antisense oligonucleotides, dominant-negative mutants, ribozymes, small interfering RNAs, cancer vaccines for immunotherapy [100], and small-molecule inhibitors [101,102]. So far, many reviews on small-molecule survivin inhibitors have been reported [101,102,103]. In this review, we describe a more updated set of new small-molecule survivin inhibitors as potential anticancer agents, as listed in Figure 4 and Table 1. We categorize them in this review, based on their putative mechanisms of action, into inhibitors that decrease survivin gene transcription, inhibitors that disrupt survivin homodimerization, and inhibitors that disrupt survivin’s interactions with its partner proteins. 

### 4.1. Inhibitors That Decrease Survivin Gene Transcription

#### 4.1.1. YM155 (**1**)

A small molecule survivin inhibitor YM155 (**1**) was first discovered via the high-throughput screening (HTS) of the in-house large chemical compound libraries owned by Astellas Pharma (Japan) in 2007 [104]. By using a survivin promoter–luciferase reporter system transfected in cervical carcinoma HeLa cells, Nakahara et al. identified YM155 as a small molecule that significantly inhibits survivin expression at both the mRNA and protein levels when used at 10–100 nM levels [104]. YM155 potently inhibited survivin-promoter-driven luciferase expression without affecting the expression of cIAP2, XIAP, Bcl-2, Bcl-XL, Bad [104], cIAP1, p53, or STAT3 [105], even up to 100 nM. However, YM155 also inhibits Mcl-1 expression in prostate cancer PC-3, mesothelioma H28, glioblastoma U251, and D37 cancer cells [106]. Another mechanism by which YM155 inhibits survivin expression involves inhibiting the survivin upstream transcription factor, specificity protein 1 (Sp1), and disrupting its interaction with the region of -149 to -71 in the survivin core promoter [107]. Moreover, it has been shown that YM155 inhibits survivin expression by disrupting the transcription factor, interleukin enhancer-binding factor 3 (ILF3) and the p54nrb complex, which binds to the survivin promoter and regulates the expression of survivin [108]. Furthermore, recent studies suggest Topoisomerase (TOP) but not survivin as the molecular target of YM155 [109]. Therefore, it is unlikely that YM155 exerts its anticancer effects by inhibiting solely survivin expression. Preclinically, YM155 effectively induced tumor regression in human prostate PC-3 ectopic xenograft tumors without causing body weight loss [104]. The combination of YM155 with alemtuzumab, an anti-CD52 monoclonal antibody, in a murine model of human adult T-cell leukemia (ATL) significantly prolonged the survival of tumor-bearing mice, and all the mice that received the combination therapy survived and were tumor-free > 6 months after treatment [110]. In xenograft models, the continuous infusion of four cycles of YM155 eradicated large, established subcutaneous WSU-DLCL-2 diffuse large cell lymphomas and Ramos tumors and significantly increased survival vs. rituximab, an anti-surface protein molecule cluster of the differentiation-20 (CD20) monoclonal antibody [111]. A YM115 and rituximab combination treatment induced significant tumor growth inhibition and tumor regression compared with either single agent in human B-Cell non-Hodgkin’s lymphoma xenografts [112]. Moreover, continuous infusion of YM155 decreased survivin expression, reduced metastases, and significantly prolonged survival in a murine model of spontaneous metastatic human triple-negative breast cancers (TNBCs) [113]. Along with breast cancer (MDA-MB-231), continuous infusions of YM155 demonstrated significant antitumor activity in non-small cell lung cancer (NSCLC) (Calu 6 and NCI-H358), melanoma (A375), and bladder cancer (UM-UC-3) xenograft models without showing significant body-weight loss [114]. Furthermore, YM155 was also shown to increase the sensitivity of human NSCLC to gamma-radiation in tumor xenografts in nude mice [105]. Additional preclinical studies indicated that a combination treatment of YM155 and docetaxel, a tubulin inhibitor, also induced a greater rate of apoptosis than the sum of the single-treatment rates and promoted tumor regression without significant toxicity, as indicated by the little body-weight loss that was recorded in the melanoma xenograft models [115]. Moreover, the combination of YM155 and cisplatin induced apoptosis and tumor regression in cisplatin-resistant head and neck squamous cell carcinoma (HNSCCs) [116] and ovarian cancer cells [117]. Moreover, YM155 potentiated chemosensitivity to gemcitabine in pancreatic cancer MiaPaCa2 cells’ xenograft tumors [118]. Despite the favorable outcome of YM155 in preclinical studies and its good tolerability with a maximum tolerated dose (MTD) of 4.8 mg/m^2^ [119], multiple phase I and phase II studies using YM155 either as a single agent or in combination with other cytotoxic therapeutic agents demonstrated that YM155 exhibits very limited antitumor efficacy [120,121,122,123,124,125]. Many reasons could be responsible for the failure of YM155 in recent clinical trials. Firstly, the uncertainty of the molecular target of YM155 is an obstacle against bringing a drug to clinical oncology. Secondly, YM155 must be given via a continuous infusion 24 h a day in 3- or 7-day dosing cycles because of its short half-life (YM155 plasma concentrations after the i.v. bolus injection is 1.06 h) [104]. Lastly, YM155 is a substrate of the P-glycoprotein (P-gp); hence, multidrug resistance could be a liability in its clinical use [126]. For example, it has been shown that the expression of MDR1 (also known as ABCB1) predicts the resistance of neuroblastoma (NB) cells to YM155 [127].

#### 4.1.2. FL118 (**2**)

In 2012, Ling et al. discovered FL118 (**2**) through HTS of compound libraries. FL118 is a nonselective small-molecule inhibitor of survivin expression [128]. FL118 shows potent inhibition of survivin promoter activity, survivin expression, and cancer cell growth at high pM to low nM concentrations [128]. It also inhibits the expression of other cancer-associated IAPs, such as Mcl-1, XIAP, and cIAP2. FL118 is a structurally camptothecin (CPT) analog and a topoisomerase I (Top1) inhibitor; however, the concentration required for FL118 to show its Top1 inhibition activity is 100- to 1000-fold higher than the concentration needed for FL118 to inhibit both survivin promoter activity and cancer cell growth [101,128,129]. Compared to other CPT analogs such as irinotecan, SN-38 (the active metabolite of irinotecan), and topotecan, which are substrates of the efflux pump proteins ABCG2/BCRP [167,168] and P-gp/MDR1 [169,170], FL118 is not a substrate of ABCG2 or P-gp and can overcome treatment resistance resulting from the expression of ABCG2 [130] or P-gp [131]. The superior antitumor efficacy of FL118 depends on its steric configuration and the presence of a free hydroxyl group at position 20. FL118, which has an R- configuration, showed superior anticancer potency compared with FL113, the racemic mixture of FL118. Both FL118 and FL113 completely lost antitumor activity in vivo upon esterification of the hydroxyl group t position 20 [132]. Recently, Li et al. reported that DDX5 (also known as p68) could be the direct target of FL118, and they showed that FL118 binds to and inhibits both the phosphorylation and expression of DDX5 in colorectal cancer (CRC) and pancreatic ductal adenocarcinoma (PDAC) cancer [133]. DDX5 is an upstream master regulator in cancer and positively controls the expression of survivin, Mcl-1, XIAP, and cIAP2 [171]. Furthermore, Li et al. demonstrated that FL118 degrades the DDX5 in pancreatic cancer Mia Paca-2 and Panc-1 cells and colorectal cancer SW620 cells through the ubiquitin–proteasome degradation pathway, without decreasing the DDX5 mRNA level [133]. FL118 has shown potent antitumor activity in patient-derived tumor xenografts in animal models such as human head and neck tumor xenograft models [128] and human colon tumor xenografts [132]. Moreover, FL118 alone or combined with gemcitabine effectively eliminated tumors in pancreatic cancer patient-derived xenograft (PDX) animal models [134]. In their recent review, Li et al. indicated that FL118 would proceed into clinical trials with the indication of colorectal and pancreatic cancers in a year or so [101].

#### 4.1.3. SF002-96-1 (**3**)

SF002-96-1 was identified and isolated from Aspergillus by Felix et al. using a survivin-promoter-driven reporter assay in the colorectal cancer cell line Colo320 [135]. In addition to the inhibition of survivin-promoter-driven luciferase activity in a dose-dependent manner with an IC_50_ of 3.42 μM, SF002-96-1 inhibited STAT3-dependent luciferase expression with an IC_50_ value of 1.6 μM. SF002-96-1 also inhibited NF-κB-dependent reporter gene expression with an IC_50_ value of 2.63 μM. STAT3 and NF-κB are involved in the regulation of the survivin expression; thus, SF002-96-1 could inhibit survivin expression by inhibiting multiple upstream transcription factors [135].

#### 4.1.4. Terameprocol (Also Known as EM-1421, M4N, **4**)

Terameprocol was synthesized based on 3′-O methylnordihydroguaiaretic acid (NDGA), a plant lignan isolated from Larrea tridentate that suppresses human immunodeficiency virus type 1 (HIV-1) replication in infected human cells [172]. Terameprocol selectively inhibits Sp1 and Sp1-dependent cyclin-dependent kinase (Cdc2), survivin, and vascular endothelial growth factor (VEGF) expression and induces growth arrest and apoptosis [136,137]. Preclinically, Park et al. established that terameprocol, when given systemically, can safely and effectively suppress in vivo growth of human tumor xenografts, including liver, prostate, colorectal, and breast cancers [138]. Furthermore, the combination of terameprocol and everolimus (RAD001) significantly reduced insulin-like growth factor binding protein 2 (IGFBP-2) overexpression and synergistically suppressed endometrial cancer growth [139]. In phase I/II clinical trials, terameprocol showed an excellent safety profile and partial responses in patients with advanced leukemia, cervical intraepithelial neoplasia, and recurrent high-grade glioma [140,141,142].

#### 4.1.5. WM-127 (**5**) 

WM-127 was identified based on matrine, an alkaloid found in plants from the genus of Sophora Flavescens, using a survivin-promoter/regulatory-sequence-driven EGFP (Sur5P-EGFP-Sur3U) reporter system in the hepatocellular carcinoma (HCC) HepG2 cell line [143]. WM-127 inhibited survivin protein and delayed HCC xenograft tumors in nude mice. Yin et al. showed that WM-127 might function by inhibiting the activity of the survivin/β-catenin pathway and increasing the expression of the Bax protein [143].

#### 4.1.6. GDP366 (**6**)

GDP366 was identified using a small-scale compound in-house library. Shi et al. showed that in human colon cancer HCT116 cells, GDP366 inhibited the gene and protein expression of both survivin and stathmin 1 (STMN1, also known as an oncoprotein 18 (Op18)), a microtubule destabilizing phosphoprotein. In vivo, GDP366 inhibited the HCT116 xenograft mouse model growth without any significant toxicity [144]. However, a contradictory report showed that survivin expression was increased after GDP366 treatment in AML and acute lymphoblastic leukemia (ALL) [145].

### 4.2. Inhibitors That Disrupt Survivin Homodimerization

#### 4.2.1. Abbot 8 (**7**) and Its Analogs

Abbot 8 was identified by Abbot Laboratories using NMR- and affinity-based screening of their libraries for compounds binding to survivin [146]. Abbot 8 was reported to bind at the dimerization interface of survivin. Computational modeling of the molecular interactions of Abbot 8 along the survivin dimerization interface led to the design of LLP3 (**8**) and LLP9 (**9**). LLP3 and LLP9 modulate cell cycle progression and cause major mitotic defects including defects in CPC organization and prolonged mitosis in proliferating human umbilical vein endothelial cells (HUVEC) and prostate cancer PC-3 cells at low nanomolar concentrations [147]. Guvenc et al. reported that LLP-3 treatment disrupted the survivin–Ran protein complex, eliminated the Ran effector molecule TPX2, and abolished the growth of patient-derived glioblastoma multiforme (GBM) in vitro and in vivo [148]. Furthermore, a recent study indicated that LLP3 might be used to sensitize CRC cells to irinotecan, which depends on XAF1 proficiency in the context of mutated p53. In the same study, Steigerwald et al. demonstrated that LLP3 might also be effective as monotherapy in the subgroup of p53-proficient and some p53-mutated tumors in CRC, independent of mismatch repair status [149]. 

#### 4.2.2. S12 (**10**)

S12 was identified via computational in silico screening of the small molecules that can bind survivin in a cavity close to the dimeric interface, applying the cavity-induced allosteric modification (CIAM) approach. S12 targets a specific cavity adjacent to the survivin dimerization surfaces and induces allosteric conformational changes in the protein structure that disrupt the normal functions of survivin. S12 treatment alters spindle formation and cell cycle progression and causes accumulation of cells in the G2/M phase (similar to survivin deletion) [150]. S12 inhibited the proliferation and growth of sonic hedgehog-driven medulloblastoma cancer cells in vitro [151]. Moreover, S12 impeded the growth of pancreatic xenograft tumors in a dose-dependent manner in vivo [150]. However, it is unclear whether S12 disrupts survivin dimerization or affects survivin interaction with its binding partners [150]. 

#### 4.2.3. Indinavir (**11**) and Nelfinavir (**12**)

Indinavir, an HIV protease inhibitor, was identified as a potential compound that could target survivin protein–protein interactions (PPI). It was identified by Sarvagalla et al., who first detected hot spots residues in the survivin dimer and its binding partner CPC, then derived a pharmacophore model, and used it to virtually screen database compounds [152]. Treatment of breast cancer MDA-MB-231 cells with indinavir resulted in Aurora B and XIAP downregulation and caspase-3 activation, which are the hallmarks of survivin PPI inhibition [152]. Another HIV protease inhibitor, Nelfinavir, was previously shown to be able to decrease levels of the survivin protein in combination therapy with imatinib when used on primary meningioma cells and meningioma cell lines IOMM-Lee and CH157. In fact, combination therapy was found to be more effective than imatinib alone in vivo [153]. However, more studies are needed to determine whether these HIV protease inhibitors bind to and inhibit the interaction of survivin with its ligands. 

#### 4.2.4. LQZ-7 (**13**) and Its Analogs 

LQZ-7 was discovered through a combination of detailed computational analysis and in silico screening of 200,000 compounds to target the survivin dimerization interface, followed by in vitro and cell-based assays. LQZ-7 dissociated dimeric survivin by targeting the residues Leu98 and Phe101 in the dimerization interface of survivin, causing exposure of the hydrophobic dimerization core and inducing subsequent proteasome-dependent survivin degradation without affecting survivin mRNA [154]. LQZ-7F (**14**) is a lead compound, obtained through further analysis of LQZ-7 analogs, which can more effectively inhibit the survival of multiple human cancer cell lines with low micromolar IC_50_ (0.4–4.4 μM), suppress prostate PC3 xenograft tumors growth in vivo, and disrupt survivin dimerization. LQZ-7F can directly bind to the interface for survivin homodimerization, as evident by the pull-down assay with immobilized LQZ-7F and purified survivin [154]. LQZ-7I (**15**) is another analog of LQZ-7 that lacks the labile hydrazone linker and has a quinoxaline ring instead of the furazanopyrazine in LQZ-7. It effectively inhibited prostate cancer PC-3 xenograft tumor growth and reduced survivin level in vivo [155]. 

### 4.3. Inhibitors That Disrupt Survivin Interactions with its Partner Proteins

#### 4.3.1. Shepherdin (**16**)

Shepherdin is a peptidomimetic agent with a sequence K79-L87 (KHSSGCAFL) of survivin. It interrupted the interaction of Hsp90 with survivin through the ATP-binding pocket of Hsp90 and destabilized survivin [156]. Shepherdin can also destabilize several additional client proteins of Hsp90, such as Akt, CDK6, and CDK4. Shepherdin inhibited the growth of cervical carcinoma HeLa, prostate cancer PC3, and adenocarcinoma DU145 cells by inducing apoptosis, without any apparent effect on normal cells. Shepherdin also maintained its excellent antitumor activity in vivo and effectively inhibited the growth of prostate cancer PC3 xenograft tumors [156]. The use of Shepherdin significantly increased the cytotoxic activity of hydroxyurea and doxorubicin on imatinib mesylate-resistant chronic CML [157].

#### 4.3.2. AICAR (**17**)

AICAR is a nonpeptidic small molecule inhibitor that mimics the chemical and conformational properties of shepherdin. Massimiliano Meli et al. reported that shepherdin was used as a scaffold to build a three-dimensional pharmacophore to screen a database of nonpeptidic structures. Similar to shepherdin, AICAR exhibited excellent anti-proliferative activities in multiple cancer cell lines, including DU145, HeLa, and melanoma JR8 cells, without affecting the proliferation of normal human fibroblasts. It also disrupted multiple Hsp90 client proteins, including survivin [158].

#### 4.3.3. Deazaflavin Analog Compound 1 (**18**)

Deazaflavin analog compound 1 is a compound structurally related to 5-deazaflavin that disrupts the survivin–Smac interaction and replaces Smac in cancer cells. It was discovered through the HTS system of an in-house chemical library of more than 30,000 small molecules using the in vitro AlphaScreen assay [159]. It preferentially inhibits the interaction of survivin with Smac with an IC_50_ = 2.2 μM but not INCENP (IC_50_ = 20 μM) in vitro and in a culture cell system. Moreover, it was able to sensitize DU145 and lung carcinoma A549 cultured cells to doxorubicin-mediated DNA damage stress and synergistically enhance apoptotic cell death [159].

#### 4.3.4. UC-112 (**19**) and Its Analogs 

UC-112 was identified by our group in 2004 through a shape-based virtual screening against a drug-like compound library using the bioactive conformation of AVPI tetrapeptide in the N-terminus of Smac as a template [160]. UC-112 significantly induced the activation of caspases 3, 7, and 9 in melanoma and prostate cancer cell lines. Furthermore, it dose-dependently inhibited survivin expression as well as the expression of other IAPs, albeit to a lesser extent, in most of the cancer cell lines that were tested. MG-132, a pan proteasome inhibitor, can counteract the ubiquitin-mediated degradation and rescue survivin from the action of UC-112, suggesting that UC-112 may produce its survivin inhibition effect, at least in part, via the ubiquitin-mediated degradation of survivin. In vivo, UC-112 showed potent tumor growth inhibition in a melanoma A375 xenograft model, with little reduction in the body weight of the mice. A follow-up structure–activity relationship study identified MX-106 (**20**), a UC-112 analog with an isopropyl group substitution on the C-ring [161]. MX-106 was about four-fold more active than UC-112 (2.2 μM for UC-112 vs. 0.5 μM for MX-106; average GI_50_ values over all cancer cell lines in the NCI-60 panel). MX-106 exhibited increased selectivity to survivin compared with UC-112. Additionally, it effectively suppressed the growth of human melanoma A375 xenograft tumors and strongly induced cancer cell apoptosis in tumor tissues. Moreover, MX-106 sensitized TNBC tumors to doxorubicin in vivo [162]. Moreover, MX106 efficiently inhibited primary tumor growth in ovaries and metastasis in multiple peritoneal organs in an orthotopic ovarian cancer mouse model [163]. However, further optimization of the MX106 structure led to the discovery of compound 12b (**21**), which is equipotent to MX106 but more metabolically stable than MX106 (i.e., the metabolic stability of 12b improved over MX-106 by 1.7-fold (88 vs. 51 min in human microsomes)) [164]. Other UC-112 analogs have been reported by our group (10f, 10h, 10k, and 10n, as shown in Figure 4 **22**–**25**), which showed similar activity to UC-112 and maintained their unique selectivity against survivin over other IAPs [165].

#### 4.3.5. PZ-6-QN (**26**)

PZ-6-QN was identified by Park et al. through an initial screening of a library of compounds containing phenothiazine derivatives, by using the fluorescence anisotropy (FA) assay and then conducting a structure–activity relationship study. PZ-6-QN, which contains a quinolinium cation as a mitochondria-targeting motif, disrupts the interaction of survivin with Smac in mitochondria. Based on cell-based mechanistic studies, Park et al. proposed that PZ-6-QN enters mitochondria to block the survivin–Smac interaction and, in turn, promotes the release of Smac and Cyt-c from mitochondria into the cytosol. PZ-6-QN exhibits good anticancer activity against various cancer cells, including HeLa, A549, colon cancer HCT-116, and breast cancer cell MCF-7, with IC_50_ values ranging between 2.0 and 4.0 μM [166].

## 5. Final Remarks: Survivin in Cancer and the Efforts for Targeting Survivin So Far

The interest in survivin biology is foreseeable because survivin distinguishes itself by its preferential expression in most human tumor cells. Moreover, survivin expression has been positively correlated with increased tumor resistance to radiation and chemotherapy, and it promotes tumor cell survival and cancer metastasis through its roles in apoptosis inhibition, mitosis, autophagy, and angiogenesis. Even though our understanding of survivin has expanded exponentially, only a small number of small-molecule survivin inhibitors were reported in the last decade. This is because targeting survivin for direct binding affinity is challenging, since survivin lacks intrinsic catalytic activity and has no druggable site. Most small-molecule survivin inhibitors do not directly target survivin; instead, they are dependent on the inhibition of the survivin signaling pathway. They reduce survivin expression or activity by suppressing the transcription or translation of survivin and its functionally related molecules. This may explain the lack of experimental co-crystal structures of survivin proteins in complexes with true small molecule inhibitors in the protein databank, as the 43 currently available crystal structures are either wild-type survivin itself from different species, mutant survivin itself, or survivin in complexes with a variety of peptide segments. Therefore, it is difficult to evaluate the efficacy of small-molecule survivin inhibitors and their on-target or off-target toxicity; hence, there is an observed discrepancy between the promising preclinical data of small-molecule survivin inhibitors and their unsatisfactory performance in phase I/II clinical trials. Moreover, the inhibition of the survivin signaling pathway could be bypassed by compensatory upregulation of other pathways involving IAP family proteins or the induction of different isoforms of survivin. Going forward, the strategy should be to identify synergistic combination treatments or dual inhibitors. Such an approach could lead to dose reductions, minimize toxicity, and optimized therapy for patients.

## Figures and Tables

**Table 1:** Small-molecule survivin inhibitors reviewed in this article.

Inhibitor	Mechanism of Action	Current Status	References
YM155 (**1**)	Inhibits survivin expression at both mRNA and protein levels; inhibits the survivin upstream transcription factors, Sp1 and ILF3, and their interactions with survivin promoter	Phase II; combination with docetaxel as first-line treatment for HER2-negative metastatic breast cancer; completed. Phase II; combination with rituximab for CD20-positive B cell non-Hodgkin’s lymphoma; completed Phase II; combination with docetaxel for stage III (unresectable) or stage IV melanoma; completed Phase II; alone for stage III (unresectable) or metastatic (stage IV) melanoma; completed. Phase II; combination with paclitaxel and carboplatin for advanced non-small cell lung carcinoma; completed Phase II; combination with docetaxel and prednisone for advanced hormone-refractory prostate cancer and other solid tumors; completed Phase II; alone for relapsed/refractory c-Myc rearranged high-grade B-cell lymphoma; recruiting	[104,105,106,107,108,109,110,111,112,113,114,115,116,117,118,119,120,121,122,123,124,125,126,127]
FL118 (**2**)	Inhibits survivin expression at both mRNA and protein levels	Preclinical	[128,129,130,131,132,133,134]
SF002-96-1 (**3**)	Inhibits survivin expression by inhibiting STAT3 and NF-κB	Preclinical	[135]
Terameprocol (**4**)	Inhibits survivin expression	Phase I; for intravenous administration in leukemia; completed Phase I; for intralesional injection in refractory malignant tumors of the head and neck; completed Phase I/II; for intravenous infusion administration in recurrent high-grade glioma; completed. Phase I; for oral administration in recurrent high-grade glioma; active Phase I/II; for intravaginal administration in cervical intraepithelial neoplasia induced by human papillomavirus; completed	[136,137,138,139,140,141,142]
WM-127 (**5**)	Inhibits survivin expression	Preclinical	[143]
GDP366 (**6**)	Inhibits survivin gene and protein expression	Preclinical	[144,145]
Abbot 8 (**7**), LLP3 (**8**), and LLP9 (**9**)	Disrupt survivin dimerization	Preclinical	[146,147,148,149]
S12 (**10**)	Disrupts survivin dimerization	Preclinical	[150,151]
Indinavir (**11**) and Nelfinavir (**12**)	Target survivin protein–protein interactions	Approved for HIV infection	[152,153]
LQZ-7 (**13**), LQZ-7F (**14**), and LQZ-7I (**15**)	Dissociate dimeric survivin and induce subsequent proteasome-dependent survivin degradation	Preclinical	[154,155]
Shepherdin (**16**)	Disrupts survivin interactions with Hsp90 and destabilizes survivin	Preclinical	[156,157]
AICAR (**17**)	Disrupts survivin interactions with Hsp90 and destabilizes survivin	Preclinical	[158]
Deazaflavin analog compound 1 (**18**)	Inhibits the interaction of survivin with Smac	Preclinical	[159]
UC-112 (**19**), MX-106 (**20**), Compound 12b (**21**), 10f (**22**), 10h (**23**), 10k (**24**) and 10n (**25**)	Increase the ubiquitin-mediated degradation of survivin	Preclinical	[160,161,162,163,164,165]
PZ-6-QN (**26**)	Disrupts the interaction of survivin with Smac in mitochondria	Preclinical	[166]

## Data Availability

Not applicable.

## Abbreviations

AIF, apoptosis-inducing factor; AIP, aryl hydrocarbon receptor-interacting protein; ALL, acute lymphoblastic leukemia; AML, acute myeloid leukemia; Apaf-1, apoptotic protease activating factor-1; APC/C, anaphase-promoting complex or cyclosome; ATL, adult T-cell leukemia; BIR, baculovirus IAP repeat; BIRC5, baculoviral inhibitor of apoptosis repeat-containing 5; CDE, cell-cycle dependent element; CHR, cell-cycle gene homology region; CIAM, cavity-induced allosteric modification; CIAP-1, cellular inhibitor of apoptosis protein 1; CIAP-2, cellular inhibitor of apoptosis protein 2; CML, chronic myeloid leukemia; CPC, chromosomal passenger complex; CPT, camptothecin; CRC, colorectal cancer; Crm1, chromosome region maintenance 1; Cyt-c, cytochrome c; FA, fluorescence anisotropy; GBM, glioblastoma multiforme; HBXIP, hepatitis B virus X-interacting protein; HCC, hepatocellular carcinoma; HIV-1, human immunodeficiency virus type 1; HNSCCs, head and neck squamous cell carcinoma; Hsp90, heat shock protein 90; HTS, high-throughput screening; HUVEC, human umbilical vein endothelial cells; IAPs, inhibitor of apoptosis proteins; IGFBP-2, insulin-like growth factor binding protein 2; ILF3, interleukin enhancer-binding factor 3; IMS, intermembrane space of mitochondria; INCENP, inner centromere protein; MAPs, microtubule-associated proteins; MOMP, mitochondrial outer membrane permeability; MTD, maximum tolerated dose; MTS, mitochondrial targeting sequence; NB, neuroblastoma; NDGA, nordihydroguaiaretic acid; NES, nuclear export sequence; NLS, nuclear localization signal; NSCLC, non-small cell lung cancer; PDAC, pancreatic ductal adenocarcinoma cancer; PDX, patient-derived xenograft; P-gp, P-glycoprotein; RanBP1, RanGTP-binding protein 1; RanGAP, RanGTPase-activating protein; RanGEF, Ran guanine nucleotide exchange factor; SAF, spindle assembly factor; Smac, second mitochondria-derived activator of caspases; Sp1, specificity protein 1; STMN1, stathmin 1; TNBCs, triple-negative breast cancers; Top1, topoisomerase I; TOP, topoisomerase; TPX2, targeting protein for Xenopus kinesin-like protein 2; VEGF, vascular endothelial growth factor; XIAP, X-linked inhibitor of apoptosis protein.
